# Diagnosis and treatment of coronary spasm in China: a case report

**DOI:** 10.3389/fcvm.2024.1398675

**Published:** 2024-08-15

**Authors:** Hongyang Zhang, Xianglin Ye, Haifeng Pei

**Affiliations:** ^1^ Department of Cardiology, The Affiliated Hospital, Southwest Medical University, Luzhou, China; ^2^ Department of Cardiology, The General Hospital of Western Theater Command, Chengdu, China

**Keywords:** coronary vasospasm, myocardial bridging, angina, coronary angiography, intracoronary ultrasound

## Abstract

**Background:**

Coronary vasospasm (CVS) is a common cardiovascular condition, yet its implications should not be underestimated. Regrettably, the current diagnostic and treatment standards for CVS in China are not standardized, severely affecting the quality of life for patients with this condition.

**Case presentation:**

A 68-year-old male presented to the hospital one month prior due to recurrent chest pain. Coronary angiography (CAG) revealed a mid-segment muscle bridge with plaque formation in the left anterior descending artery, followed by pharmacological balloon angioplasty. The primary diagnosis post-operation was acute non-ST elevation myocardial infarction (NSTEMI) and coronary artery myocardial bridging. This time, the patient experienced nocturnal chest pain with a dynamic increase in troponin levels. Emergency CAG showed the left anterior descending and right coronary arteries were fine, with segmental narrowing reaching 95%–99%. Intravascular ultrasound (IVUS) indicated negative remodeling of the mid-segment lumen associated with myocardial bridging, with the smallest lumen area being 2.19 mm^2^. After intracoronary administration of nitroglycerin, the original most narrowed lumen area increased to 8.81 mm^2^. Consequently, a definitive diagnosis of CVS with coronary artery myocardial bridging was made, and the medication treatment plan was promptly adjusted. The patient's symptoms disappeared, and he was discharged. Follow-up after more than three months showed no recurrence of symptoms.

**Conclusion:**

In cases where provocative agents are contraindicated, CAG combined with IVUS can optimize the differential diagnosis of CVS. There is an urgent need in China to improve epidemiological data on CVS and establish standardized diagnostic and treatment protocols.

## Key points

• When there are contraindications to the use of provocation tests, the combination of coronary angiography (CAG) and intravascular ultrasound (IVUS) can optimize the differential diagnosis of coronary vasospasm (CVS);
• Myocardial bridging (MB) is one of the significant factors contributing to the exacerbation and recurrence of CVS symptoms;
• Drug misuse is another significant factor contributing to the exacerbation and recurrence of CVS symptoms;
• The current state of prevention and treatment for CVS in China is not optimistic, urgently necessitating the improvement of comprehensive epidemiological studies and the formulation of localized expert consensus/guidelines.

## Background

Coronary vasospasm (CVS) is a reversible constriction spontaneously occurring in the smooth muscle of the epicardial coronary arteries, which can occur in both angiographically normal vessels and areas of plaque stenosis, leading to partial or complete occlusion of the vessel and associated with various myocardial ischemic syndromes (1, 2). Clinically, CVS typically presents as episodic chest pain without an apparent trigger, accompanied by transient ST-segment changes on the electrocardiogram, with a minority of patients experiencing acute myocardial infarction or even sudden death due to persistent severe coronary artery spasm. Although diagnostic testing for CVS reactivity has been proven effective (3), provocation tests are still infrequently performed in clinical practice, especially since the 2013 revision of the Japanese Circulation Society (JCS) guidelines identified confirmed myocardial ischemic injury as a contraindication (Class III) (4). Moreover, due to the atypical clinical manifestations of CVS, timely and definitive diagnosis remains a significant challenge. Furthermore, medication misuse can easily lead to CVS recurrence, severely impacting the patient's quality of life and even life safety. Epidemiological studies indicate that, compared to Caucasians, Asian patients are more susceptible to coronary vasospasm, a trend particularly pronounced among the Japanese population (5, 6). The prevalence of the condition has led Japanese scholars to conduct more in-depth research on CVS and to develop corresponding treatment and diagnostic guidelines (7), while China still lacks localized treatment protocols and comprehensive epidemiological surveys for CVS patients. Considering the potential differences among ethnicities, this work could contribute to the optimization of treatment protocols for CVS patients in China.

## Case presentation

A 68-year-old male was evaluated in the emergency department for repetitive episodes of chest discomfort. The patient reported that the chest pain began 2 months prior without any apparent cause, radiating to the throat and accompanied by a sensation of chest compression, tightness of breath, and profuse sweating. Each incident had a duration of roughly 10 min and subsided without medical intervention. He did not seek medical attention or treatment. He disclosed a prolonged history of nicotine and ethanol use exceeding five decades, yet had discontinued nicotine use and curtailed ethanol consumption in the recent 10 months. Moreover, he was diagnosed with hypertension nine months ago, receiving sacubitril/valsartan sodium tablets treatment, which yielded positive outcomes.

**Figure 1:**
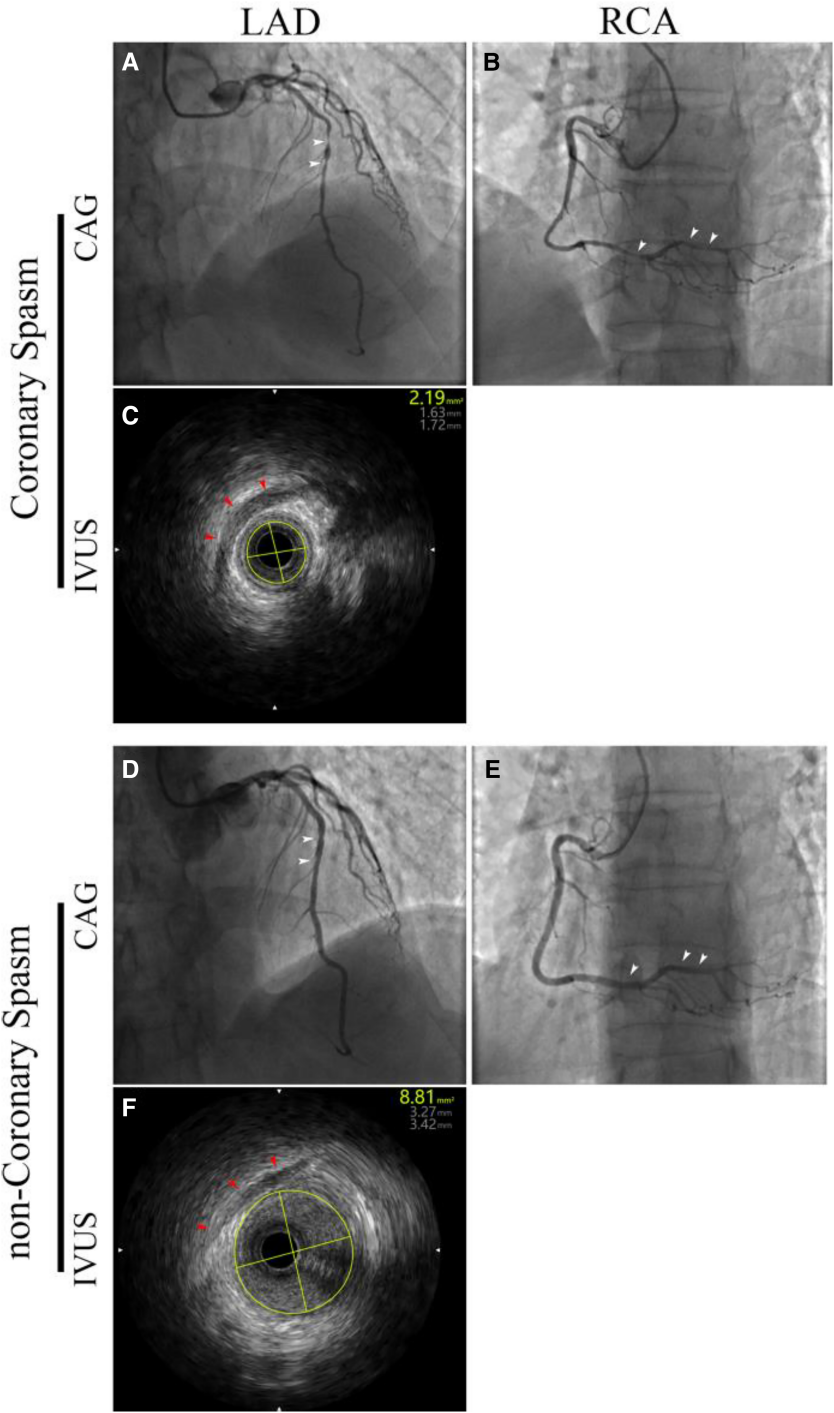
Emergency coronary angiography (CAG) and intravascular ultrasound (IVUS) were conducted in response to a recurrence of chest pain one day ago. During coronary spasm: CAG showed **(A)** the entire segment of the left anterior descending (LAD) artery was slender, with mid-segment narrowing of 95%–99% (white arrow), and **(B)** the entire segment of the right coronary artery (RCA) was slender, with part of the segment narrowing 95%–99% (white arrow); **(C)** myocardial bridging (red arrow), with the minimum luminal area of the LAD at 2.19 mm^2^. During non-coronary spasm: **(D)** the entire segment of the LAD was significantly dilated compared to before, with segmental narrowing resolved (white arrow); **(E)** the entire segment of the RCA was significantly dilated compared to before, with segmental narrowing disappeared (white arrow); **(F)** follow-up IVUS showed that the luminal area at the initially narrowest point had recovered to 8.81 mm^2^.

Approximately one month earlier, the individual presented with an episode of chest discomfort that extended to the throat and left shoulder, manifested by paraesthesia and muscular weakness in the upper extremities, which spontaneously ameliorated after nearly 50 min. Subsequently, the individual sought care at our outpatient facility and was hospitalized. The electrocardiographic evaluation revealed (See Supplementary Figure S1A): (1) Sinus rhythm at 88 beats per min; (2) Minor ST segment elevation; (3) T-wave anomalies. The concentration of high-sensitivity troponin I was measured at 0.238 ug/ml (reference limit 0.058 ug/ml), B-type natriuretic peptide (BNP) at 12.82 pg/ml (reference limit 100 pg/ml), and low-density lipoprotein cholesterol (LDL-C) at 3.38 mmol/L (acceptable range: 1.50–3.30 mmol/L). With continuing symptoms of chest discomfort post-admission, further assessment after 2 h indicated a rise in high-sensitivity troponin I to 0.390 ug/ml. Urgent coronary angiography was undertaken, revealing: (1) Unobstructed flow in the left main coronary artery and circumflex artery, with TIMI grade 3 perfusion; (2) A muscular bridge along with plaque accumulation causing 95% stenosis in the mid-segment of the left anterior descending artery, with TIMI grade 2–3 flow; (3) The right coronary artery exhibited no stenosis, with TIMI grade 3 flow. Percutaneous transluminal coronary angioplasty (PTCA) with a drug-eluting balloon was executed at the stenotic site within the left anterior descending artery, followed by a subsequent angiographic assessment revealing residual stenosis below 20% (See Supplementary Figure S2). Following a 48 h period of vigilant observation devoid of any episodes of chest discomfort, the patient was discharged with a dual diagnosis of: (1) Acute Non-ST Elevation Myocardial Infarction (NSTEMI); (2) Myocardial bridging of the coronary artery. It was recommended that the patient commence a regimen comprising aspirin, ticagrelor, rosuvastatin, β-blockers, Azilsartan and isosorbide mononitrate sustained-release capsules. In the early nocturnal hours of the preceding day, the patient reported an abrupt, persistent constrictive discomfort in the left pectoral region, persisting for approximately 30 min, with symptoms similar to previous episodes. Electrocardiographic analysis disclosed (See Supplementary Figure S1B): (1) Sinus rhythm, 74 beats per min; (2) Slight elevation of the ST segment and T-wave changes in leads V1–V5. Serial measurements of high-sensitivity troponin I levels demonstrated an escalation: 0.052 ug/ml upon emergency presentation, 1.146 ug/ml four hours post-admission, culminating at 4.333 ug/ml seven hours post-admission. Emergency coronary angiography demonstrated: (1) Absence of stenosis in the left main trunk, the left anterior descending (LAD) artery was slender with mid-segment 95%–99% narrowing (Figure 1A), with TIMI grade 2–3 blood flow; (2) The circumflex artery was slender with TIMI grade 3 blood flow; (3) No stenosis observed in the right coronary artery, with TIMI grade 3 blood flow; (4) The posterior lateral branch was slender with proximal 95%–99% stenosis (Figure 1B), with TIMI grade 2–3 blood flow. Intravascular ultrasound (IVUS) of the left anterior descending (LAD) branch reveals: negative remodeling of the mid-LAD lumen with concurrent coronary artery myocardial bridging, presenting a minimal luminal area of 2.19 mm^2^ (Figure 1C). Subsequent intracoronary administration of 100 μg nitroglycerin led to a re-evaluation angiogram which revealed: (1) Mid-segment myocardial bridge of the LAD, with 50% compression of the LAD during systole, and TIMI grade 3 blood flow (Figure 1D); (2) No narrowing in the posterior lateral branch, with TIMI grade 3 blood flow (Figure 1E). Follow-up IVUS of the LAD indicated the mid-segment myocardial bridging, with the original narrowest lumen area restored to 8.81 mm^2^ (Figure 1F). The postoperative diagnosis was: (1) Coronary vasospasm; (2) Coronary artery myocardial bridging. After the condition stabilized, a complete echocardiogram (ECHO) indicated: (1) Mild regurgitation of the mitral and tricuspid valves; (2) Normal left ventricular systolic function (LVEF 60%). The patient was advised to take a calcium channel blocker (diltiazem hydrochloride sustained-release capsules) to prevent coronary artery spasms, discontinue beta-blockers and long-acting nitrates, and add short-acting nitrate medication for temporary use during acute angina attacks. The patient was discharged after the condition stabilized. Over a three-month follow-up period, the patient reported no recurrence of discomfort.

## Discussion

In the case at hand, the patient experienced chest pain early in the morning one day prior without any apparent triggering factors. The electrocardiogram at admission revealed elevated ST segments in leads V1–V5. Emergency coronary angiography indicated severe stenosis, amounting to 95%–99%, in the mid-segment of the left anterior descending artery and the proximal segment of the posterior lateral branch (Figure 1A, highlighted by white arrows). To determine if the observed stenosis was attributable to atherosclerotic plaque formation, intravascular ultrasound imaging (IVUS) was expedited. The IVUS indicated negative remodeling at the site of stenosis, with the concomitant presence of a myocardial bridge, and a minimum lumen area of 2.19 mm^2^. Considering the patient had undergone drug-eluting balloon angioplasty a month prior, the progression of the lesion should not have been so rapid, leading to the analysis that coronary vasospasm (CVS) was the most likely cause. Following the intracoronary administration of 100 μg nitroglycerin and a subsequent coronary angiogram (CAG) review, the severe stenosis in the mid-segment and proximal segment of the left anterior descending artery and posterior lateral branch disappeared (Figures 1D,E, indicated by white arrows). A follow-up intravascular ultrasound (IVUS) in the left anterior descending artery showed that the narrowest part of the lesion had increased to 8.81 mm^2^. The current reliable method for diagnosing Coronary Vasospasm (CVS) involves inducing spasms with provocative agents (such as acetylcholine or ergonovine) during coronary angiography and considering an observation of coronary artery lumen narrowing >90% as a positive result (7). However, conducting this test during coronary angiography is deemed risky in the presence of confirmed myocardial injury (categorized as Class III in the 2013 revised JCS guidelines) (4). In the most recent edition of the guidelines, the evidence level has been elevated to Class IIb. However, this presupposes the initial definitive exclusion of other conditions such as plaque rupture and spontaneous coronary artery dissection (SCAD) (7). The patient in this case belongs to this high-risk group, as indicated by the dynamic increase in troponin levels suggesting significant myocardial ischemic injury. In this case, the IVUS examination significantly aided in the determination of the CAG results, conclusively diagnosing CVS and effectively guiding pharmacological treatment by medical personnel. This indicates that, when the use of provocative agents is contraindicated, combining CAG with IVUS can improve the differential diagnosis of coronary conditions. Although coronary artery spasm was assisted in diagnosis by IVUS during the second hospitalization, it is still recommended to conduct a provocative test for strict diagnosis after the condition stabilizes, to further ensure the standardization of medication. Additionally, if the IVUS had been performed during the first hospitalization, the team could have identified the possibility of coronary artery spasm in this patient earlier, provided a definitive diagnosis, and administered the correct treatment plan. However, in the medical process, the doctor is responsible for formulating the treatment plan, while the final decision on the choice of the plan depends on the patient and their family. Reviewing the situation at that time, the examination plan was clearly refused after being communicated to the patient and their family. Additionally, literature reports indicate that for patients with recurrent angina pectoris who are found to have complete stenosis during CAG examination, intracoronary nitroglycerin injection can be performed before intervention if there are no risk factors (8). In this case, if this procedure had been performed after stenosis was observed during the first emergency CAG, the spasm might have been promptly resolved. However, IVUS is still required for definitive imaging diagnosis because atherosclerotic plaque rupture may also be accompanied by coronary spasm.

The occurrence of CVS involves endothelial dysfunction and localized hyperreactivity, leading to an inappropriate increase in vascular smooth muscle contractility, which results in partial or complete occlusion of the coronary arteries. It is noteworthy that in this patient, two areas within the range affected by the left anterior descending myocardial bridging (MB) exhibited spasmodic narrowings more severe than those in other vessels (Figure 1A, indicated by white arrows). Traditionally, myocardial bridging (MB) has been perceived as a benign anatomical variant, primarily because it leads to restricted coronary blood flow during systole with its minimal effects during diastole being neglected (9). A retrospective analysis highlighted that in patients with non-obstructive coronary artery disease, myocardial bridging (MB) is associated with CVS occurring at the same site, with a significantly higher incidence than in patients without MB (10). This association is speculated to stem from the prolonged influence of continuous contraction-relaxation cycles on the vessel wall at the MB site, precipitating alterations in endothelial functionality (11). Therefore, for angina patients observed with “milking sign” (a typical imaging manifestation of myocardial bridging) during CAG examination, the possibility of concurrent CVS should be considered. In the absence of contraindications, conducting coronary reactivity testing may serve as a better method for identifying high-risk patients (12).

According to the current Japanese guidelines, long-acting calcium channel blockers (CCBs) are advocated as both a preventive and therapeutic intervention for CVS (Class I), while short-acting nitrates are recommended for alleviating spasms during episodic attacks (7). On the other hand, the clinical application of long-acting nitrates is discouraged due to the development of tolerance from extended usage. Although β-blockers can reduce myocardial oxygen demand by diminishing blood flow perfusion, their propensity to trigger CVS via α-receptor stimulation renders them unsuitable for exclusive use in CVS patients lacking significant fixed coronary stenosis. In the established treatment regimen for myocardial bridging, β-blockers and calcium channel blockers (CCBs) are predominantly suggested, as they can mitigate symptoms by decreasing cardiac contractility, thus reducing coronary compression (9). Considering this patient's simultaneous presentation of CVS, myocardial bridging, and hypertension, a thorough analysis suggests that calcium channel blockers (CCBs) should be the favored option. Reviewing the patient's medical history, the initial hospitalization was diagnosed as acute non-ST segment elevation myocardial infarction and coronary myocardial bridging, for which β-blockers and isosorbide mononitrate sustained-release capsules (long-acting nitrates) were prescribed. This treatment approach might be one of the causes for the exacerbation and recurrence of chest pain within a month for the patient. After the second hospitalization, which definitively diagnosed CVS, the treatment plan was adjusted to long-term administration of diltiazem hydrochloride sustained-release capsules. Follow-up of the patient for over three months showed no recurrence of the condition. Research indicates that nitroglycerin intake can increase vascular compliance, enhancing the compression of the coronary lumen by MB during systole and potentially worsening symptoms (13). This evidence suggests that long-term use of β-blockers and long-acting nitrates in treating patients with concurrent CVS and MB might provoke adverse reactions, necessitating cautious application.

The experience of diagnosing and treating this case serves as an alert to healthcare professionals about the high incidence of missed diagnoses and medication misuse among Chinese CVS patients. This issue results in uncontrollable or recurrent conditions, adversely affecting patients’ quality of life and safety, and significantly squandering national healthcare resources. Therefore, an exhaustive epidemiological study of CVS patients in China is imperative, and the establishment of a consensus and guidelines by Chinese CVS experts is urgently needed.

## Data Availability

The original contributions presented in the study are included in the article/Supplementary Material, further inquiries can be directed to the corresponding author.
