# Supplementary material for: Diagnosis and treatment of coronary spasm in China: a case report
Source: Front Cardiovasc Med. 2024 Aug 15;11:1398675. doi: 10.3389/fcvm.2024.1398675 (PMC11363188; doi:10.3389/fcvm.2024.1398675)
Supplement: Supplementary file 1 [file Datasheet1.pdf]

Supplementary Figures:

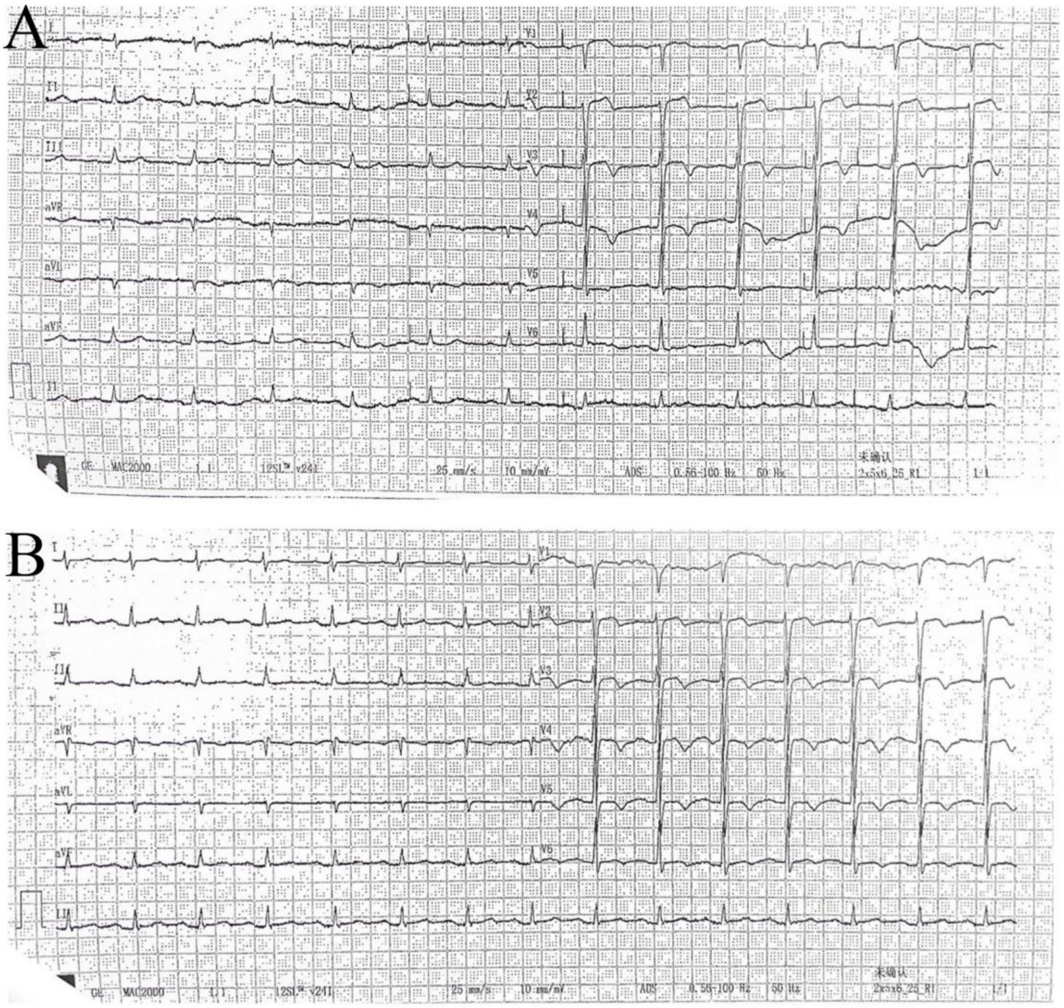

Supplementary Figure 1. Electrocardiogram on two admissions.

(A) The first admission electrocardiographic evaluation revealed: 1. Sinus rhythm at 88 beats per minute; 2. Minor ST segment elevation; 3. T-wave anomalies. (B) The second admission electrocardiographic evaluation revealed: 1. Sinus rhythm, 74 beats per minute; 2. Slight elevation of the ST segment and T-wave changes in leads V1-V5.

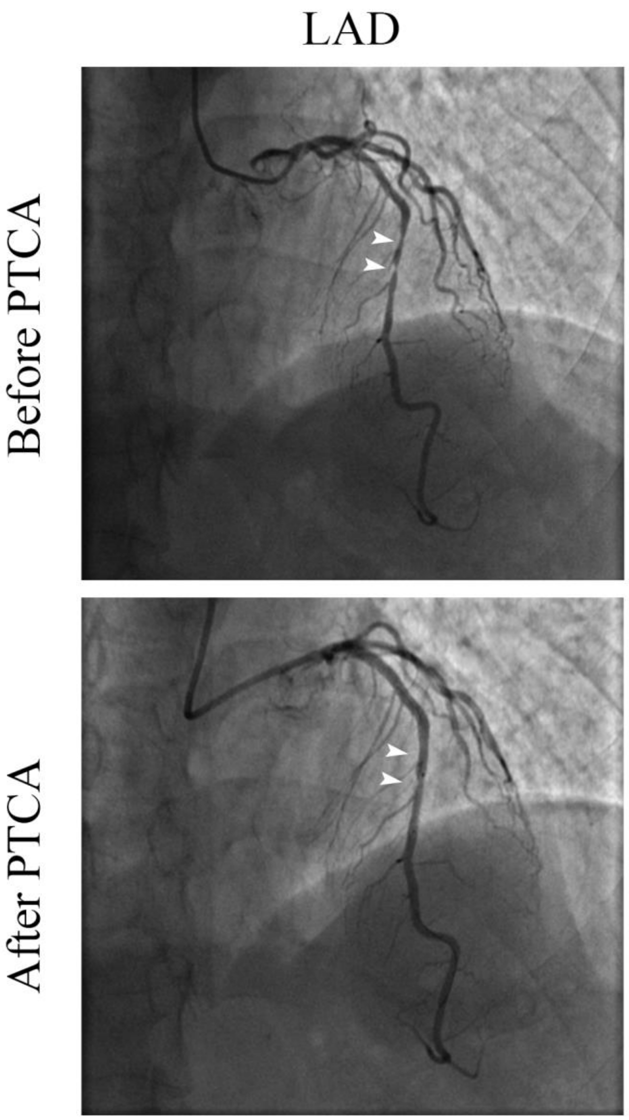

Supplementary Figure 2. Before and after effects of percutaneous transluminal coronary angioplasty (PTCA) of the left anterior descending (LAD) artery at the first admission. Percutaneous transluminal coronary angioplasty (PTCA) with a drug-eluting balloon was executed at the stenotic site within the left anterior descending artery, followed by a subsequent angiographic assessment revealing residual stenosis below 20%.
